# A comparison of two sleep spindle detection methods based on all night averages: individually adjusted vs. fixed frequencies

**DOI:** 10.3389/fnhum.2015.00052

**Published:** 2015-02-17

**Authors:** Péter Przemyslaw Ujma, Ferenc Gombos, Lisa Genzel, Boris Nikolai Konrad, Péter Simor, Axel Steiger, Martin Dresler, Róbert Bódizs

**Affiliations:** ^1^Institute of Behavioral Science, Semmelweis UniversityBudapest, Hungary; ^2^Department of General Psychology, Pázmány Péter Catholic UniversityBudapest, Hungary; ^3^Centre for Cognitive and Neural Systems, University of EdinburghEdinburgh, UK; ^4^Department of Clinical Research, Max Planck Institute of PsychiatryMunich, Germany; ^5^Department of Cognitive Sciences, Budapest University of Technology and EconomicsBudapest, Hungary; ^6^Nyírõ Gyula Hospital, National Institute of Psychiatry and AddictionsBudapest, Hungary; ^7^Donders Institute for Brain, Cognition and Behaviour, Radboud University Medical CentreNijmegen, Netherlands

**Keywords:** EEG, sleep spindles, sigma waves, automatic detections, fixed frequency method, IAM, comparison

## Abstract

Sleep spindles are frequently studied for their relationship with state and trait cognitive variables, and they are thought to play an important role in sleep-related memory consolidation. Due to their frequent occurrence in NREM sleep, the detection of sleep spindles is only feasible using automatic algorithms, of which a large number is available. We compared subject averages of the spindle parameters computed by a fixed frequency (FixF) (11–13 Hz for slow spindles, 13–15 Hz for fast spindles) automatic detection algorithm and the individual adjustment method (IAM), which uses individual frequency bands for sleep spindle detection. Fast spindle duration and amplitude are strongly correlated in the two algorithms, but there is little overlap in fast spindle density and slow spindle parameters in general. The agreement between fixed and manually determined sleep spindle frequencies is limited, especially in case of slow spindles. This is the most likely reason for the poor agreement between the two detection methods in case of slow spindle parameters. Our results suggest that while various algorithms may reliably detect fast spindles, a more sophisticated algorithm primed to individual spindle frequencies is necessary for the detection of slow spindles as well as individual variations in the number of spindles in general.

## Introduction

Sleep spindles are oscillations emerging from interacting thalamocortical, corticothalamic, and reticular networks in NREM sleep (Steriade and Deschenes, 1984; Amzica and Steriade, 2000; Steriade, 2000; Fogel and Smith, 2011), which are thought to play an important role in sleep-related brain plasticity (Genzel et al., 2014). Due to their trait-like nature and relationship to plasticity, sleep spindles are frequently studied as candidate indexes of individual variations in cognitive performance. Sleep spindles are remarkably individual features: sleep spindle parameters are characterized by high intra-individual stability and inter-individual variability (De Gennaro et al., 2005), a strong genetic background (De Gennaro et al., 2008), and a correlation with anatomical properties of the brain (Piantoni et al., 2013; Saletin et al., 2013).

Due to their high prevalence and specific signal properties automatic detection methods have proven to be viable and preferable alternatives to visual detection. Some of the earliest studies (Broughton et al., 1978; Campbell et al., 1980) used phase-locked loop devices for automatic sleep spindle detection and already reported an adequate agreement with visual detection. An early combined software-hardware system (Ferri et al., 1989) also reliably replicated visual spindle detection results. Software solutions for automatic spindle detection were introduced somewhat later (Schimicek et al., 1994) and reported relatively high (approx. 70%) specificity for 90% sensitivity, while an improved method (Devuyst et al., 2006) could increase this to almost 76% in a clinical sample. More recently, sophisticated automatic sleep spindle detection methods using artificial neural networks (Acır and Güzeliş, 2004; Ventouras et al., 2005) and decision trees (Duman et al., 2009) reached even higher performance, with correct classification frequently exceeding 90%.

Automatic sleep spindle recognition was further refined by adapting algorithms that take into account the inter-individual differences in sleep spindle activity, which vastly exceed intra-individual variation (De Gennaro et al., 2005) and emerge—among others—as a function of age and sex (Driver et al., 1996; Carrier et al., 2001; Huupponen et al., 2002; Genzel et al., 2012). Sleep spindle detection methods have been developed to operate with individually adjusted amplitude limits (Huupponen et al., 2000, 2007; Ray et al., 2010). A novel algorithm (Bódizs et al., 2009; Ujma et al., 2014) based on the electrophysiological fingerprint theory of human sleep (De Gennaro et al., 2005, 2008) is the Individual Adjustment Method (IAM), which takes into account inter-individual variations not only in the amplitude, but also in the frequency of sleep spindles. In the IAM, sleep spindles are therefore not only detected based on individual amplitude thresholds, but also within the exact frequency bands where they are present in a given individual. A similarly adaptive detection method (based on a probabilistic model) is reported in Nonclercq et al. (2013).

A comparison of four different spindle detection methods (Huupponen et al., 2007) reported acceptable, but not overwhelming concordance. A recent study (Warby et al., 2014) investigated the agreement in spindle detection between expert human raters, non-experts recruited in an internet crowdsourcing effort, and automatic detection algorithms. Concordance was strongest among human experts, followed by non-experts operation in a crowdsourcing scheme, and weakest among automatic algorithms.

While the progress in automatic sleep spindle detection methods is impressive, there are numerous concerns which must be addressed in this field. A practical criticism may arise from the fact that automatic sleep spindle detections are frequently validated against visual detections: however, agreement in the visual scoring of spindles is not perfect (Campbell et al., 1980; Warby et al., 2014), the visual detection of spindles is often considered as a consensus from several raters which may bias results (Ray et al., 2010), and—despite stronger agreement among human raters than algorithms (Warby et al., 2014)—the use of human expert opinion as an absolute gold standard is philosophically questionable in itself (Bódizs et al., 2009).

Further criticism must be given to the fact of the use of standard signal detection terminology (such as sensitivity and specificity) in case of sleep spindle detection algorithms. Sleep spindles are frequent phenomena, but even so the vast majority of a sleep EEG recording does not consist of sleep spindles. Therefore, correct negative classifications are by far the most common result produced by any sleep spindle detector, which might drastically inflate specificity. The ratio of correct hits and false detections—including misses and false positives—would be a much more conservative, but also more informative measure of detection performance.

Sleep spindles are not only biological signals, but important markers of individual traits (De Gennaro et al., 2005, 2008) as well as powerful correlates of human cognition (among others: Bódizs et al., 2005; Schabus et al., 2006; Fogel et al., 2007; Ujma et al., 2014). Therefore, an alternative option in order to assess detection algorithms would be to investigate how much they can reproduce trait-like individual averages (instead of comparing individual spindle detections).

To our knowledge, it has never been investigated how strongly spindle measures of different detection methods are correlated if not individual spindle detections, but subject averages are considered. This can evidently not predicted from the signal detection characteristics of the comparison of individual spindle detections of various methods—albeit the literature usually reports moderate agreement between the individual spindle detections of different algorithms, it is unknown whether the different spindle samples obtained by different methods approximate the same individual averages. Therefore, the aim of our study was to reveal the correlation between individual sleep spindle parameters calculated with two different detection methods.

## Materials and methods

### Subjects

We examined polysomnographic data of 161 healthy volunteers (88 males, 73 females, age between 17 years and 69 years, mean age 29.4 years, StD 10.7 years) recorded on the second night spent in a sleep laboratory. All procedures were approved by the responsible institution's ethical board and subjects gave informed consent. A semi-structured interview excluded any history of neurologic or psychiatric disease, but six subjects suffered from frequent nightmares. Subjects were free of drugs and prescription medication (except for contraceptives, all data self-reported). Alcohol and excessive caffeine consumption (over two cups of coffee before noon) was not allowed. Eight subjects were smokers, while the rest were non-smokers (self-reported). This dataset used for analysis was the same as in Ujma et al. (2014), except for the inclusion of one female subject who was excluded from the previous study due to her unavailable IQ score.

### Sleep recordings

All subjects spent two nights in a sleep laboratory and polysomnographic data from the 2nd night was used for analysis. Since the study was performed in cooperation between multiple sleep laboratories, recordings were performed in four slightly different designs.

For 31 subjects, recordings were performed with 18 EEG electrodes using a Flat Style SLEEP La Mont Headbox device with a HBX32-SLP preamplifier (La Mont Medical Inc. USA), with a sampling rate of 249 Hz, hardware prefiltering 0.5–70 Hz and a precision of 12 bit.

For 16 subjects signals were collected, prefiltered (0.33–1500 Hz, 40 dB/decade anti-aliasing hardware input filter), amplified and digitized with 4096 Hz/channel sampling rate (synchronous) and 12 bit resolution by using the 32 channel EEG/polysystem (Brain-Quick BQ 132S, Micromed, Italy). A further 40 dB/decade anti-aliasing digital filter was applied by digital signal processing which low-pass filtered the data at 450 Hz. Finally, the digitized and filtered EEG was undersampled at 1024 Hz.

For 114 subjects, recordings were performed with a Comlab 32 Digital Sleep Lab device (Schwarzer, Germany) with a sampling rate of 250 Hz, hardware prefiltering 0.53–70 Hz and a precision of 8 bit. In 94 of these subjects, 22 EEG electrode sites were used, while in the others 20 subjects 10 EEG electrodes were used. Common recording sites in all subjects which were used in the analysis were Fp1, Fp2, F3, F4, Fz, F7, F8, C3, C4, Cz, P3, P4, T3, T4, T5, T6, O1, and O2, all referred to the mathematically linked mastoids. For the 20 subjects with only 10 electrodes, data from Fp1, Fp2, F3, F4, C3, C4, P3, P4, O1 and O2 was available and for the other electrodes these subjects were treated as missing data.

In order to correct for potentially different baseline amplitudes depending on the recording device (Vasko et al., 1997), the analog-digital conversion and filtering characteristics of all recording devices were measured and sleep spindle amplitudes were corrected for the measured differences as follows (Ujma et al., 2014). We determined the amplitude reduction rate of each recording system by calculating the proportion between digital (measured) and analog (generated) amplitudes of sinusoid signals at typical sleep spindle frequencies (10, 11, 12, 13, 14, and 15 Hz) for both inducing (40 and 355 μV amplitude) signals. Machine-specific amplitude reduction rates were given as the mean amplitude rate between digital and analog values at the two amplitudes and six measured frequencies. Sleep spindle amplitudes were corrected by dividing their calculated values by the amplitude reduction rate of the recording system. Given the individual- and derivation-specific adjustment inherent to both the Fixed frequency method (FixF) and the IAM, sleep spindle densities and durations are amplitude-insensitive measures. Thus, there is no need for the compensation of the different recording systems in these values.

Sleep recordings of the second nights were scored according to standard criteria (Iber et al., 2007) on a 20 s basis and artifacts were removed by visual inspection on a 4 s basis. Sleep spindle analysis was performed on artifact-free segments of NREM sleep.

### Analyses

#### Fixed frequency method of sleep spindle analysis

For the FixF method we determined the 11–13 Hz range as a slow spindle frequency band and the 13–15 Hz window as a fast spindle frequency band. These frequencies were selected to ensure consistency with previous studies (Schabus et al., 2006, 2007, 2008; Chatburn et al., 2013), which used a similar approach for the separation of slow and fast spindles.

Sleep spindles were automatically detected within artifact-free NREM sleep periods on every EEG derivation. For slow spindle detection, data were bandpass-filtered between 11 Hz and 13 Hz. The root mean squares of the filtered signals were determined for 0.25 s length time windows. Next a threshold was calculated at the 95th percentile of the root mean square values for every EEG derivation. A spindle was identified when at least two consecutive root mean square time points exceeded the threshold, and the duration criterion (≥0.5 s) was met. Four spindle characteristics were calculated; these were density (number of spindles/min); amplitude (peak-to-peak difference in voltage, expressed in μV); duration (s), and frequency (number of cycles/s, in Hz). The same procedure was followed for detecting fast spindles, using a band pass filter of 13–15 Hz (Schabus et al., 2007; Gruber et al., 2013).

#### Sleep spindle analysis according to the IAM

The second sleep spindle detection algorithm was the IAM (Bódizs et al., 2009). This sleep spindle detection method takes into account both inter-individual variations and intra-individual consistency in sleep spindle frequency (De Gennaro et al., 2005, 2008), analyzing sleep spindles at the individual peak frequency for all subjects.

**Figure 1:**
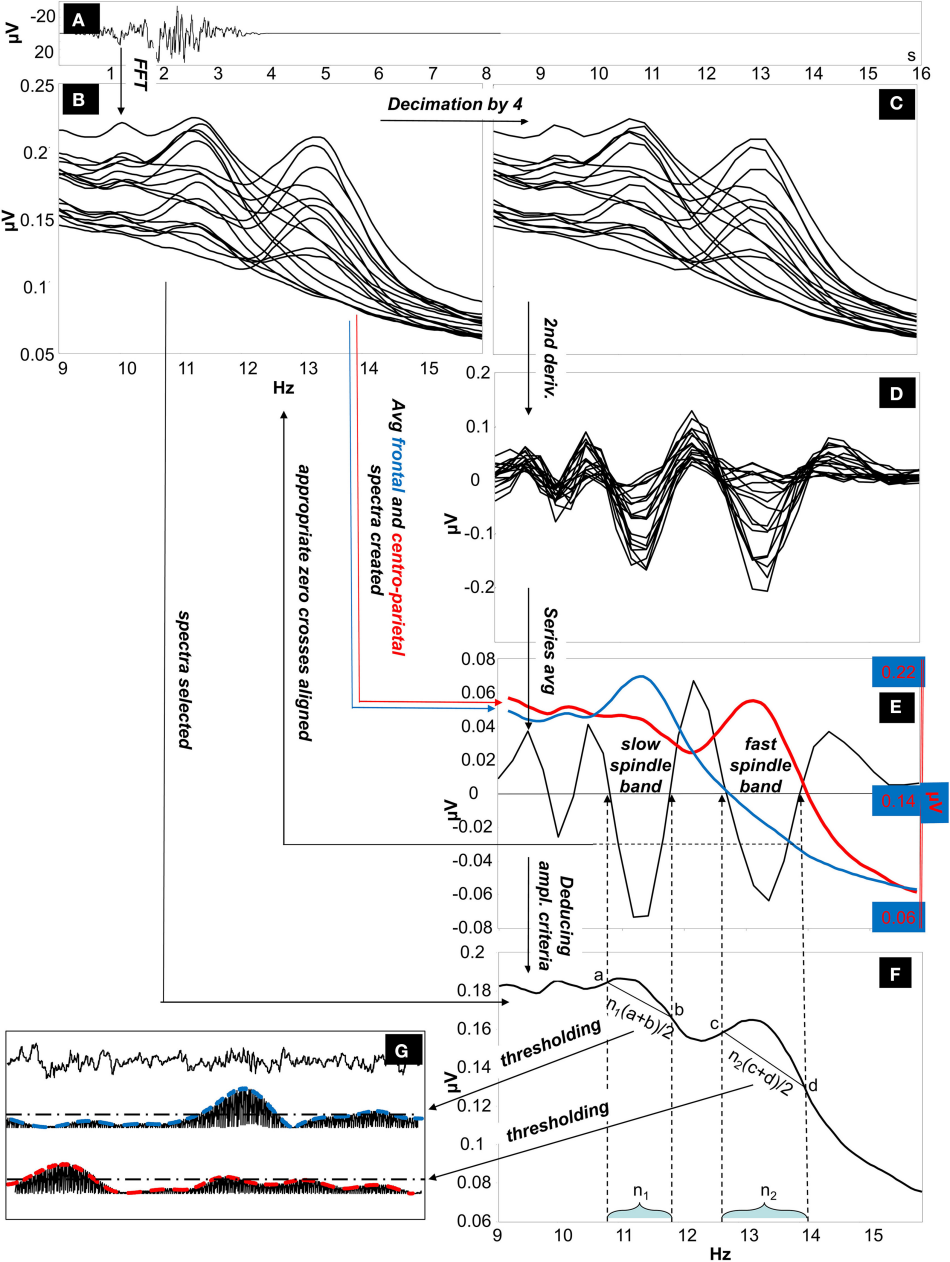
**The Individual Adjustment Method (IAM) of sleep spindle analysis**. **(A)** Four-second EEG epoch Hanning-tapered and zero padded to 16 s. **(B)** Fast Fourier Transformation (FFT) is used to calculate 9–16 Hz average amplitude spectra of all night NREM sleep EEG from Hanning-tapered and zero-padded segments (derivations: Fp1, Fp2, F3, F4, Fz, F7, F8, T3, T4, T5, T6, C3, C4, Cz, P3, P4, O1, O2 referred to the mathematically-linked mastoids). **(C)** Amplitude spectra are decimated (down-sampled) by a factor of 4. **(D)** Second order derivatives of the decimated amplitude spectra. **(E)** Calculating the whole-scalp second order derivatives by averaging all series. The resulting average series is overplotted with the averaged frontal (Fp1, Fp2, F3, F4, Fz, F7, F8) and centro-parietal (C3, C4, Cz, P3, P4) amplitude spectra (the left-side Y axis is for average second-order derivatives, while the second Y axis on the right is for average amplitude spectra). Appropriate zero-crossing points encompassing individual-specific slow and fast sleep spindle bands are selected on the 9–16 Hz frequency scale. **(F)** Derivation-specific amplitude criteria are calculated. **(G)** Thresholding of the envelopes of the slow and fast-spindle filtered signal.

The IAM procedure (Bódizs et al., 2009) consisted of several steps as described below (illustrated on Figure 1).

- Average amplitude spectra. Non-overlapping 4 s artifact-free NREM sleep EEG segments are Hanning-tapered (50%), then zero-padded to 16 s. Average amplitude spectra of all-night NREM sleep EEG derivations is computed between 9 Hz and 16 Hz by using an FFT routine (frequency resolution: 0.0625 Hz).
- Individually adjusted frequency limits of slow and fast sleep spindles. Determination of the individual slow and fast sleep spindle frequencies is based on second order derivatives of the 9–16 Hz amplitude spectra. In order to avoid small fluctuations in convex and concave segments average amplitude spectra of 0.0625 Hz resolution (i) is downsampled (decimated) by a factor of 4 (resulting in a resolution of 0.25 Hz) before calculating the derivation-specific second-order derivatives in this frequency range. Derivation-specific second order derivatives of the amplitude spectra are then averaged over all EEG derivations resulting in a whole-scalp second order derivative for each subject. Individual-specific frequency limits of sleep spindles are defined as pairs of zero crossing points encompassing a negative peak in the whole-scalp second order derivatives. These zero-crossing points are rounded to the closest bins within the high-resolution (0.0625 Hz) amplitude spectra obtained in step i. Two pairs of individual-specific frequency limits and corresponding ranges are defined (one for slow and one for fast spindles). In cases of uncertainty (lack of zero crossing points indicating slow spindles or partial overlap between slow and fast sleep spindles in some cases), frequencies with predominance of power in averaged frontal (Fp1, Fp2, F3, F4, Fz, F7, F8) over averaged centro-parietal (C3, C4, Cz, P3, P4) amplitude spectra were considered as slow spindle frequencies (*N* = 18). There was no case of uncertainty related to the individual-specific frequency boundaries of fast sleep spindles.
- Individual-specific spindle middle frequencies. Slow spindle middle frequency of a given subject was quantified as the arithmetic mean of the individual-specific lower and upper limits for slow spindling as obtained above (ii). In case of fast sleep spindling the arithmetic mean of the lower and the upper frequency limits of fast sleep spindles were considered.
- Individual- and derivation-specific amplitude criteria for sleep spindles. Spindles are defined as those EEG segments contributing to the peak region of the average amplitude spectrum. Hence we intended to obtain an amplitude criterion corresponding to the line determined by the *y*-values (μV) pertaining to the individually adjusted pairs of frequency limits (ii) in the average amplitude spectra (i).
  - The number of high resolution (0.0625 Hz) frequency bins (i) falling in the individual-specific slow- and fast sleep spindle frequency ranges (ii) is determined.
  - The amplitude spectral values (i) at the individually adjusted frequency limits for slow and fast sleep spindles (ii) are determined. This is performed in a derivation-specific manner.
  - Number of bins for slow and fast sleep spindling (iv/a) are multiplied by the arithmetic mean of the pairs of derivation-specific amplitude spectral values for slow and fast sleep spindle frequency limits (iv/b), respectively. Outcomes are individual- and derivation specific amplitude criteria for slow and fast sleep spindle detections.
- Envelopes of sleep spindling. EEG data is band-pass filtered for the slow and fast spindle frequency ranges by using an FFT-based Gaussian filter with 16 s windows: *f*(*x*) = e^(−(x− x_*m*_)/(w/2)), where x varies between zero and the Nyquist frequency according to the spectral resolution, x_*m*_ is the middle frequency of the spindle range (iii), and w is the width of the spindle range (ii) (ii and iii). Filtered signal is rectified and smoothed by a moving average weighted with a Hanning window of 0.1 s length and multiplied with π /2 (the latter is the inverse of the mean of a rectified sine wave).
- Detection and characterization of sleep spindles. If envelopes of this band-pass filtered and rectified data (v) exceed the individual and derivation-specific threshold as defined above (iv) for at least 0.5 s, a sleep spindle is detected. Sleep spindles detected this way are analyzed and average sleep spindle density (number of spindles per minute), sleep spindle duration (s), as well as median and maximum amplitude (expressed as all-night means of intra-spindle envelopes in μV at the middle of the detected spindles and at the maxima of the spindles, respectively) is calculated for the subject.

### Statistics

FixF and IAM spindle parameters were compared using paired-sample *t*-tests (α = 0.05). The Benjamini-Hochberg method of false detection rate correction was performed in order to correct for multiple comparisons.

We computed Pearson's point-moment correlation coefficients between comparable sleep spindle measures (that is, sleep spindle parameters computed from the same electrode) produced by IAM, and the FixF method.

## Results

### IAM frequency bands

For the IAM method, individual slow spindle lower frequency limits ranged from 8.98 Hz to 12.95 Hz (mean: 10.96 Hz), while higher frequency limits ranged from 10.14 Hz to 13.7 Hz (mean: 11.9 Hz). Slow spindle middle frequencies ranged from 9.59 to 13.28 Hz (mean: 11.43 Hz). Fast spindle lower frequency limits ranged from 11.82 Hz to 14.77 Hz (mean: 13.06 Hz), while higher frequency limits ranged from 13.04 Hz to 16.03 Hz (mean: 14.36 Hz). Fast spindle middle frequencies ranged from 12.49 Hz to 15.38 Hz (mean: 13.71 Hz).

Individual slow spindle frequency bands were on average 0.94 Hz wide (range: 0.34–2.2 Hz). Individual fast spindle frequency bands were on average 1.3 Hz wide (range: 0.84–1.89 Hz).

**Figure 2:**
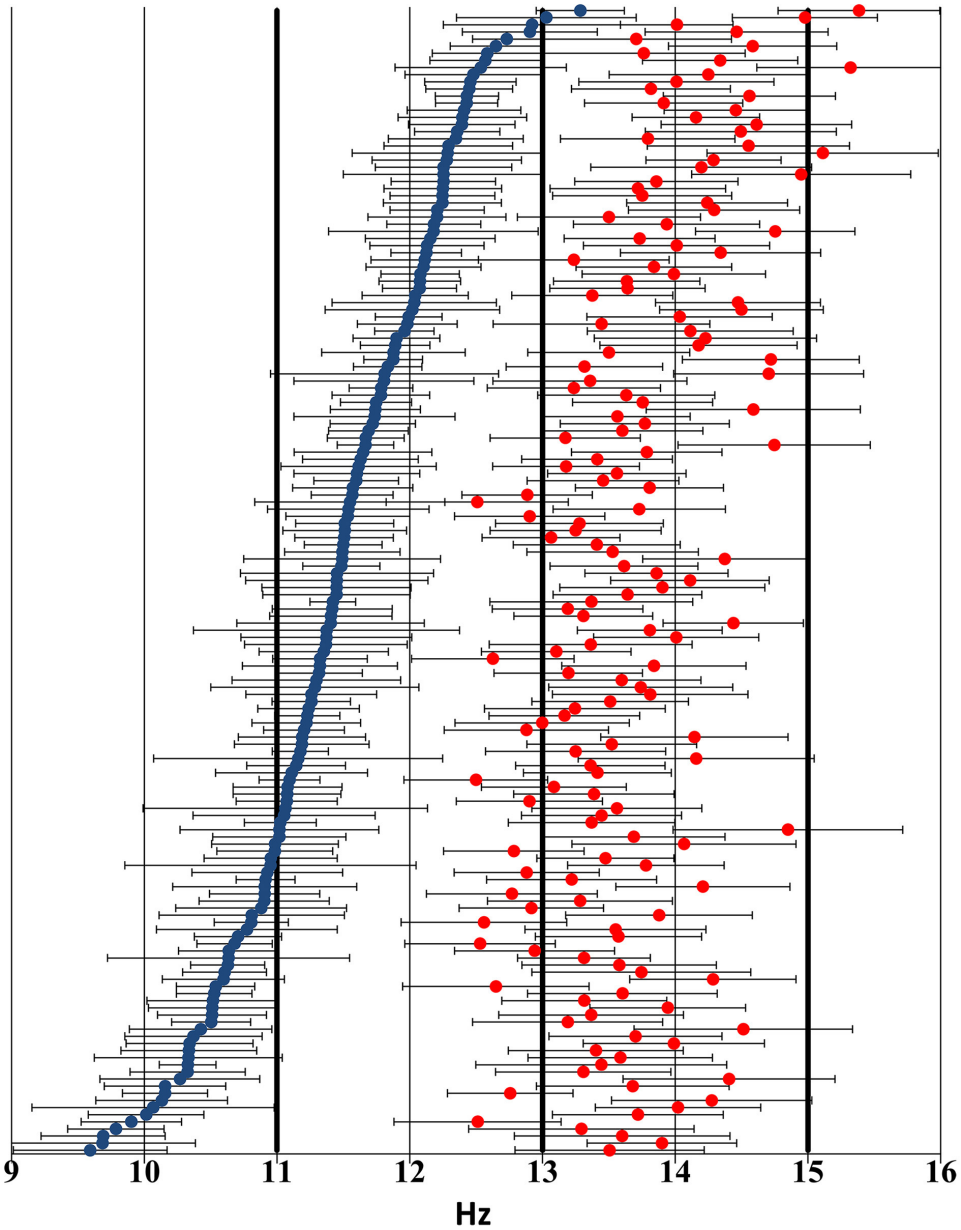
**Distribution of individual sleep spindle frequency bands across the 9–16 Hz frequency domain based on visual inspection of the sleep EEG spectrum and the zero crossings of the second-order derivatives thereof**. Blue markers indicate slow spindle middle frequencies, while red markers show fast spindle middle frequencies. Negative and positive error bars illustrate lower and higher individual frequency limits, respectively. Thick lines highlight the 11 Hz, 13 Hz, and 15 Hz thresholds used in the FixF method. Subjects have been ordered by slow spindle middle frequency to ensure better visibility.

Figure 2 shows the distribution of individual sleep spindle frequencies.

### FixF vs. IAM spindle parameters

IAM provides an approximately twice higher sleep spindle density than the FixF method in case of both slow and fast spindles as well as 1.5–2 times longer sleep spindle durations. Standard deviations of the individual averages of the FixF parameters are much smaller than in case of IAM parameters, even proportionally to the lower mean values.

Sleep spindle parameters are shown in Table 1. It must be noted that while FixF and IAM amplitude measures are displayed and compared, they are not expected to be on the same scale due to the narrower frequency band of IAM and the fact that in the FixF method amplitude was expressed as the mean maximum peak-to-peak voltage difference within a spindle, while in IAM amplitude was defined as the mean maximum of intra-spindle envelopes of the individually band-passed EEG.

**Table 1:** **Sleep spindle parameters calculated by IAM and the fixed frequency method (FixF)**.

		**FixF**	**IAM**	**FixF**	**IAM**	***t*-value**			**FixF**	**IAM**	**FixF**	**IAM**	***t*-value**
**C3**							**Fz**						
Slow spindles	*Density*	3.469	6.830	0.163	1.428	−29.663	Slow spindles	*Density*	3.586	6.876	0.143	1.245	−31.273
	*Duration*	0.673	1.413	0.045	0.467	−19.995		*Duration*	0.668	1.435	0.032	0.462	−19.755
	*Amplitude*	42.053	3.548	12.045	1.848	40.093		*Amplitude*	57.322	4.902	15.897	2.507	38.814
Fast spindles	*Density*	3.657	7.176	0.202	0.921	−47.383	Fast spindles	*Density*	3.614	6.571	0.201	1.007	−34.319
	*Duration*	0.699	1.074	0.049	0.141	−31.956		*Duration*	0.693	1.435	0.047	0.462	−19.067
	*Amplitude*	43.171	5.471	13.632	1.533	34.871		*Amplitude*	46.909	5.588	16.803	1.732	29.150
**C4**							**O1**						
Slow spindles	*Density*	3.487	6.878	0.143	1.430	−29.947	Slow spindles	*Density*	3.215	6.737	0.126	1.947	−22.905
	*Duration*	0.666	1.411	0.042	0.462	−20.348		*Duration*	0.662	1.365	0.069	0.476	−18.540
	*Amplitude*	41.946	3.638	11.001	1.831	43.583		*Amplitude*	28.171	2.460	10.093	1.406	32.013
Fast spindles	*Density*	3.662	6.878	0.190	1.430	−28.299	Fast spindles	*Density*	3.554	7.062	0.217	1.104	−39.561
	*Duration*	0.697	1.411	0.045	0.462	−19.516		*Duration*	0.703	1.073	0.052	0.146	−30.259
	*Amplitude*	44.092	5.542	13.640	1.536	35.637		*Amplitude*	30.627	4.062	11.602	1.395	28.844
**Cz**							**O2**						
Slow spindles	*Density*	3.401	6.692	0.156	1.526	−25.571	Slow spindles	*Density*	3.219	6.728	0.114	1.944	−22.866
	*Duration*	0.674	1.381	0.053	0.465	−17.976		*Duration*	0.656	1.366	0.064	0.479	−18.640
	*Amplitude*	49.867	4.211	13.568	2.094	39.630		*Amplitude*	28.348	2.479	10.497	1.377	31.006
Fast spindles	*Density*	3.688	6.692	0.182	1.526	−23.302	Fast spindles	*Density*	3.544	7.051	0.218	1.109	−39.346
	*Duration*	0.700	1.381	0.045	0.465	−17.349		*Duration*	0.699	1.066	0.051	0.142	−30.824
	*Amplitude*	58.700	7.324	18.546	2.076	32.806		*Amplitude*	29.904	3.975	10.755	1.319	30.364
**F3**							**P3**						
Slow spindles	*Density*	3.653	6.920	0.158	1.193	−34.449	Slow spindles	*Density*	3.275	6.743	0.154	1.741	−25.187
	*Duration*	0.673	1.459	0.032	0.459	−21.674		*Duration*	0.694	1.376	0.069	0.471	−18.183
	*Amplitude*	52.046	4.518	14.978	2.341	39.780		*Amplitude*	37.668	3.050	10.762	1.687	40.321
Fast spindles	*Density*	3.588	6.323	0.205	0.982	−34.597	Fast spindles	*Density*	3.619	7.506	0.206	0.932	−51.670
	*Duration*	0.691	1.014	0.044	0.117	−32.780		*Duration*	0.718	1.110	0.051	0.149	−31.496
	*Amplitude*	39.676	4.846	13.952	1.525	31.488		*Amplitude*	44.219	5.773	14.429	1.670	33.585
**F4**							**P4**						
Slow spindles	*Density*	3.661	6.966	0.149	1.182	−35.205	Slow spindles	*Density*	3.286	6.761	0.147	1.754	−25.046
	*Duration*	0.669	1.456	0.029	0.456	−21.834		*Duration*	0.683	1.371	0.068	0.473	−18.297
	*Amplitude*	53.057	4.585	15.579	2.316	39.050		*Amplitude*	36.159	2.992	10.716	1.616	38.834
Fast spindles	*Density*	3.607	6.357	0.204	0.996	−34.328	Fast spindles	*Density*	3.613	7.468	0.195	0.961	−49.894
	*Duration*	0.687	1.456	0.043	0.456	−21.289		*Duration*	0.716	1.104	0.048	0.150	−31.314
	*Amplitude*	40.907	4.945	16.694	1.541	27.218		*Amplitude*	42.701	5.532	14.211	1.646	32.967
Slow spindles	*Density*	3.661	6.953	0.164	1.318	−29.526	Slow spindles	*Density*	3.529	6.927	0.144	1.521	−26.503
	*Duration*	0.669	1.420	0.031	0.455	−19.595		*Duration*	0.645	1.388	0.031	0.470	−18.779
	*Amplitude*	35.259	3.253	9.820	1.617	38.323		*Amplitude*	26.048	2.312	8.012	1.201	34.914
Fast spindles	*Density*	3.481	5.561	0.186	1.101	−22.198	Fast spindles	*Density*	3.529	6.210	0.156	1.142	−27.718
	*Duration*	0.660	0.964	0.035	0.102	−33.518		*Duration*	0.654	0.993	0.039	0.113	−33.920
	*Amplitude*	23.331	2.998	7.859	0.875	30.642		*Amplitude*	19.469	2.529	6.010	0.702	33.362
**F8**							**T4**						
Slow spindles	*Density*	3.670	6.979	0.160	1.334	−29.352	Slow spindles	*Density*	3.525	6.929	0.145	1.546	−26.119
	*Duration*	0.665	1.418	0.028	0.456	−19.639		*Duration*	0.639	1.379	0.028	0.466	−18.911
	*Amplitude*	35.654	3.303	9.349	1.626	40.625		*Amplitude*	25.325	2.348	6.821	1.198	39.535
**F7**							**T3**						
Fast spindles	*Density*	3.479	5.534	0.207	1.099	−21.900	Fast spindles	*Density*	3.498	6.031	0.184	1.228	−24.304
	*Duration*	0.655	0.960	0.035	0.101	−34.075		*Duration*	0.647	0.985	0.037	0.117	−32.600
	*Amplitude*	23.663	3.039	7.774	0.885	31.412		*Amplitude*	19.517	2.601	6.174	0.785	32.388
**Fp1**							**T5**						
Slow spindles	*Density*	3.632	7.043	0.154	1.228	−34.960	Slow spindles	*Density*	3.333	6.753	0.139	1.808	−22.473
	*Duration*	0.674	1.448	0.029	0.455	−21.537		*Duration*	0.646	1.354	0.048	0.480	−17.495
	*Amplitude*	44.540	3.755	18.255	1.943	28.190		*Amplitude*	25.427	2.276	7.801	1.293	34.888
Fast spindles	*Density*	3.462	5.500	0.199	1.061	−23.961	Fast spindles	*Density*	3.544	6.849	0.188	1.058	−36.667
	*Duration*	0.669	0.969	0.036	0.099	−36.057		*Duration*	0.690	1.045	0.049	0.139	−28.762
	*Amplitude*	28.654	3.325	16.333	1.010	19.640		*Amplitude*	25.257	3.279	8.719	1.074	29.812
**Fp2**							**T6**						
Slow spindles	*Density*	3.644	7.064	0.152	1.238	−34.793	Slow spindles	*Density*	3.347	6.785	0.125	1.852	−22.075
	*Duration*	0.672	1.445	0.028	0.454	−21.579		*Duration*	0.638	1.348	0.042	0.475	−17.763
	*Amplitude*	44.180	3.783	17.303	1.927	29.443		*Amplitude*	24.813	2.241	8.184	1.213	32.509
Fast spindles	*Density*	3.477	5.569	0.204	1.070	−24.360	Fast spindles	*Density*	3.541	6.750	0.185	1.074	−35.098
	*Duration*	0.665	0.965	0.035	0.098	−36.612		*Duration*	0.680	1.033	0.048	0.132	−29.933
	*Amplitude*	28.325	3.345	13.607	1.031	23.228		*Amplitude*	23.782	3.108	7.491	0.876	32.665

*Density, duration and amplitude means, standard deviations (StD) and comparison t-values are shown*.

The difference between comparable FixF and IAM spindle parameters is significant in all cases at *p* < 0.0001, and all comparisons remain significant after correction for multiple comparisons.

### Correlations between FixF and IAM spindle parameters

Despite the differences in the results, individual spindle parameters obtained with the FixF and IAM methods are strongly correlated in case of the amplitude and duration of fast spindles. These correlations are always over 0.5 for amplitude and over 0.4 for duration and they are highest (>0.8 for amplitude, >0.7 for duration) in derivations where fast spindles are most prominent (central and parietal electrodes) as well as in occipital derivations. There is, surprisingly, a negative correlation between fast spindle density calculated by the IAM and the FixF method.

There is only a week concordance between FixF and IAM slow spindle parameters. There is no significant FixF-IAM correlation in case of slow spindle density and duration, and only a modest correlation in case of slow spindle amplitude (*r* < 0.5 except for F3).

Table 2 presents the Pearson correlation coefficients depicting the linear relationship between corresponding IAM and FixF spindle parameters on all electrodes.

**Table 2:** **Correlation coefficients and *p*-values between compatible sleep spindle parameters calculated by IAM and the fixed frequency method**.

**C3**	**Slow**			**Fast**			**Fz**	**Slow**			**Fast**		
	*Density*	*Duration*	*Amplitude*	*Density*	*Duration*	*Amplitude*		*Density*	*Duration*	*Amplitude*	*Density*	*Duration*	*Amplitude*
r	−0.072	0.095	0.379^*^	−0.292^*^	0.802^*^	0.788^*^	r	0.248^*^	−0.087	0.402^*^	−0.341^*^	0.737^*^	0.782^*^
p	0.364	0.230	0.000	0.000	0.000	0.000	p	0.003	0.306	0.000	0.000	0.000	0.000
**C4**	**Slow**			**Fast**			**O1**	**Slow**			**Fast**		
	*Density*	*Duration*	*Amplitude*	*Density*	*Duration*	*Amplitude*		*Density*	*Duration*	*Amplitude*	*Density*	*Duration*	*Amplitude*
r	−0.090	0.049	0.297^*^	−0.341^*^	0.785^*^	0.814^*^	r	−0.155	0.052	0.415^*^	−0.257^*^	0.793^*^	0.827^*^
p	0.255	0.541	0.000	0.000	0.000	0.000	p	0.049	0.515	0.000	0.001	0.000	0.000
**Cz**	**Slow**			**Fast**			**O2**	**Slow**			**Fast**		
	*Density*	*Duration*	*Amplitude*	*Density*	*Duration*	*Amplitude*		*Density*	*Duration*	*Amplitude*	*Density*	*Duration*	*Amplitude*
r	−0.067	0.056	0.320^*^	−0.367^*^	0.792^*^	0.842^*^	r	−0.019	0.041	0.317^*^	−0.282^*^	0.775^*^	0.804^*^
p	0.430	0.511	0.000	0.000	0.000	0.000	p	0.816	0.610	0.000	0.000	0.000	0.000
**F3**	**Slow**			**Fast**			**P3**	**Slow**			**Fast**		
	*Density*	*Duration*	*Amplitude*	*Density*	*Duration*	*Amplitude*		*Density*	*Duration*	*Amplitude*	*Density*	*Duration*	*Amplitude*
r	0.159	−0.050	0.623^*^	−0.296^*^	0.665^*^	0.720^*^	r	−0.146	0.105	0.304^*^	−0.347^*^	0.811^*^	0.849^*^
p	0.044	0.529	0.000	0.000	0.000	0.000	p	0.064	0.183	0.000	0.000	0.000	0.000
**F4**	**Slow**			**Fast**			**P4**	**Slow**			**Fast**		
	*Density*	*Duration*	*Amplitude*	*Density*	*Duration*	*Amplitude*		*Density*	*Duration*	*Amplitude*	*Density*	*Duration*	*Amplitude*
r	0.162	−0.078	0.365^*^	−0.235^*^	0.654^*^	0.754^*^	r	−0.144	0.116	0.260^*^	−0.392^*^	0.826^*^	0.855^*^
p	0.041	0.328	0.000	0.003	0.000	0.000	p	0.069	0.143	0.001	0.000	0.000	0.000
**F7**	**Slow**			**Fast**			**T3**	**Slow**			**Fast**		
	*Density*	*Duration*	*Amplitude*	*Density*	*Duration*	*Amplitude*		*Density*	*Duration*	*Amplitude*	*Density*	*Duration*	*Amplitude*
r	0.095	−0.086	0.429^*^	−0.121	0.459^*^	0.637^*^	r	0.048	−0.078	0.321^*^	−0.235^*^	0.644^*^	0.724^*^
p	0.259	0.310	0.000	0.151	0.000	0.000	p	0.567	0.359	0.000	0.005	0.000	0.000
**F8**	**Slow**			**Fast**			**T4**	**Slow**			**Fast**		
	*Density*	*Duration*	*Amplitude*	*Density*	*Duration*	*Amplitude*		*Density*	*Duration*	*Amplitude*	*Density*	*Duration*	*Amplitude*
	0.071	−0.074	0.421^*^	−0.040	0.419^*^	0.670^*^	r	−0.051	−0.059	0.331^*^	−0.037	0.547^*^	0.565^*^
	0.404	0.383	0.000	0.637	0.000	0.000	p	0.548	0.485	0.000	0.658	0.000	0.000
**Fp1**	**Slow**			**Fast**			**T5**	**Slow**			**Fast**		
	*Density*	*Duration*	*Amplitude*	*Density*	*Duration*	*Amplitude*		*Density*	*Duration*	*Amplitude*	*Density*	*Duration*	*Amplitude*
r	0.010	0.104	0.277^*^	0.052	0.349^*^	0.502^*^	r	−0.114	0.082	0.440^*^	−0.352^*^	0.767^*^	0.798^*^
p	0.901	0.191	0.000	0.512	0.000	0.000	p	0.177	0.329	0.000	0.000	0.000	0.000
**Fp2**	**Slow**			**Fast**			**T6**	**Slow**			**Fast**		
	*Density*	*Duration*	*Amplitude*	*Density*	*Duration*	*Amplitude*		*Density*	*Duration*	*Amplitude*	*Density*	*Duration*	*Amplitude*
r	0.237^*^	−0.158^*^	0.297^*^	−0.025	0.399^*^	0.526^*^	r	0.097	0.052	0.238^*^	−0.351^*^	0.703^*^	0.695^*^
p	0.002	0.046	0.000	0.753	0.000	0.000	p	0.251	0.542	0.004	0.000	0.000	0.000

*Significant correlations (after multiple comparison correction) are marked with an asterisk*.

Given that 1) our sample consisted of several datasets recorded on various EEG devices and 2) the FixF ranges we analyzed—while based on previous literature—did not correspond well to the frequency ranges computed by IAM, we re-analyzed our sample divided in subsamples as well as with different FixF ranges set with slow spindles between 10 Hz and 12.5 Hz and fast spindles between 12.5 Hz and 15 Hz. In both re-analyses, we attempted to replicate our most prominent results, and investigated fast spindle parameters on P4 and slow spindle parameters on F3. F3 was selected over Fz because of the higher availability of this electrode in the sample.

Results are similar across subsamples: that is, fast spindle density is negatively correlated; slow spindle density and duration are not correlated, slow spindle amplitude is moderately and positively correlated while fast spindle duration and amplitude are strongly and positively correlated. FixF-IAM correlations for slow spindles on F3 are as follows for density (*r*_Budapest1_ = 0.427, *p* = 0.016; *r*_Budapest2_ = −0.032, *p* = 0.908; *r*_Munich_ = 0.129, *p* = 0.086), duration (*r*_Budapest1_ = 0.086, *p* = 0.647; *r*_Budapest2_ = −0.143, *p* = 0.597; *r*_Munich_ = −0.072, *p* = 0.448) and amplitude (*r*_Budapest1_ = 0.353, *p* = 0.052; *r*_Budapest2_ = 0.498, *p* = 0.049; *r*_Munich_ = 0.519, *p* < 0.001). FixF-IAM correlations for fast spindles on P4 are as follows for density (*r*_Budapest1_ = −0.28, *p* = 0.127; *r*_Budapest2_ = −0.282, *p* = 0.291; *r*_Munich_ = −0.359, *p* < 0.001), duration (*r*_Budapest1_ = 0.844, *p* < 0.001; *r*_Budapest2_ = 0.661, *p* = 0.005; *r*_Munich_ = 0.805, *p* < 0.001) and amplitude (*r*_Budapest1_ = 0.75, *p* < 0.001; *r*_Budapest2_ = 0.798, *p* < 0.001; *r*_Munich_ = 0.861, *p* < 0.001).

Application of the new frequency bands also did not change the pattern of consistency of our methods significantly. With the 10–12.5 Hz FixF windows, FixF-IAM correlations for slow spindles on F3 are the following: *r*_density_ = 0.083, *p* = 0.292; *r*_duration_ = −0.069, *p* = 0.39; *r*_amplitude_ = 0.419, *p* < 0.001. With the 12.5–15 Hz FixF windows, FixF-IAM correlations for fast spindles on P4 are the following: *r*_density_ = −0.149, *p* = 0.06; *r*_duration_ = 0.802, *p* < 0.001; *r*_amplitude_ = 0.66, *p* < 0.001.

## Discussion

While previous studies compared sleep spindle detections between various manual and automatic methods (Huupponen et al., 2007; Warby et al., 2014), to our knowledge no previous study compared individual averages of sleep spindle parameters calculated by various methods. Moreover, comparisons of individual detections were usually performed on many spindles from a small number of subjects. We investigated the convergent validity of two well-known algorithms by correlating all-night averages of individual sleep spindle parameters in a large database of subjects. In this approach, the agreement between individual detections is admittedly less important than agreement between individual averages. Overall, our results highlight both similarities and differences in the two sleep spindle detection methods we compared, and they do not provide overwhelming evidence for the convergence of the two methods.

IAM is tuned to individual spindle frequencies as well as individual and derivation-specific amplitude limits, making it inherently more sensitive as evidenced by higher spindle density and longer duration. FixF, on the other hands, focuses on the upper 5% of the amplitude distribution of filtered EEG signals. While FixF appears to detect “the tips of the icebergs” with this approach, the fast spindles detected by FixF are able to realistically approximate the same fast spindle durations and amplitudes as the IAM. Concordance is much weaker, however, in case of slow spindle amplitude, completely absent in case of slow spindle density and duration, while a very surprising negative correlation between fast spindle densities were found. To explain these findings, some empirical tendencies must be considered.

First, while the 13–15 Hz FixF window for fast spindles was similar to the empirically determined individual frequencies of IAM fast spindles, this was not the case for the 11–13 Hz slow spindle window. Fast spindle middle frequencies were below 13 Hz in only 11.24% of all cases and over 15 Hz in 1.87% of cases, while slow spindle middle frequencies were below 11 Hz in 27.5% of all cases and over 13 Hz in 1.25% of all cases. This poor demarcation of slow spindles in the FixF method might explain why FixF slow spindle parameters correlate more strongly with IAM fast spindle parameters than IAM slow spindle parameters (FixF slow vs. IAM fast correlations on Cz: *r*_density_ = −0.092 *p* = 0.275; *r*_duration_ = 0.547 *p* < 0.001; *r*_amplitude_ = 0.603 *p* < 0.001, with similar tendencies on all electrodes, see Table 2 for correlations with IAM slow spindle parameters). This finding, together with poor agreement on density measures suggests that some FixF slow spindles may actually be classified as fast spindles by the IAM procedure and vice versa, explaining the confusion in both density measures and slow spindle parameters in general. This phenomenon is exemplified by some dissimilar findings in the field. That is, both slow and fast sleep spindle measures correlate with cognitive abilities in cases when the FixF method is used (Schabus et al., 2006, 2008), while in case of IAM fast spindles are much more stable correlates of cognitive performance (Bódizs et al., 2005, 2008; Ujma et al., 2014). It must be noted that sleep spindles are not stationary sinusoidal processes: they are known to shift frequencies (chirp). Negative spindle chirps (decreasing frequencies) have been reported in humans (Andrillon et al., 2011; Schonwald et al., 2011), while increasing spindle frequencies were reported in rats (Sitnikova et al., 2014). These frequency shifts are not large enough to eclipse the difference between slow and fast spindles (Andrillon et al., 2011) but spectral chirps arising in spindles close to the 13 Hz boundary might be large enough to make them “jump” it and be detected in the opposite category.

Second, the average width of the individual fast spindle frequency band was 1.3 Hz, while in case of slow spindles it was only 0.94 Hz. That is, our results show that individual fast spindle frequency bands rarely fell outside the 13–15 Hz range and they were generally closer to the 2 Hz window of the FixF method than slow spindle frequency bands. The fact the re-analysis with FixF bands resembling the empirically determined individual frequency bands of IAM (10–12.5/12.5–15 Hz, compare with IAM frequency bands on Figure 2) did not significantly improve concordance between the two methods suggests that the differences in individual spindle bandwidth may be even more important for the lack of concordance between the two methods than the mere whereabouts of the frequency limits. This is in line with previous results from an adaptive, probabilistic model (Nonclercq et al., 2013) which reported a similar robustness to the input frequency range.

Based on the above findings we hypothesize that the lack of consistency between FixF and IAM slow spindle parameters is caused by the above factors: IAM slow spindles are determined at a lower and narrower frequency, with a larger distance from fast spindle frequencies in the same subject. The same phenomenon might be speculated to explain the negative correlation between IAM and FixF fast spindle density: in subjects with higher numbers of fast spindles (by IAM definitions) around the 13 Hz cutoff point cross-contamination with slow spindles may have been elevated in the FixF method.

There is little consistency in the sleep spindle detection methods used in previous research literature concerning the relationship between spindles and human cognition. Not all studies about the relationship between sleep spindle parameters and individual differences in psychometric variables separated slow and fast spindles: many analyzed sleep spindles in general or spectral power from a broader frequency band (Clemens et al., 2005; Fogel and Smith, 2006; Fogel et al., 2007; Tucker and Fishbein, 2009; Lustenberger et al., 2012; Gruber et al., 2013). Most studies which specifically analyzed slow and fast spindles and their correlation with psychometric variables used a *post-hoc* classification of spindles based on their central frequency, usually with 13 Hz as the split point (Schabus et al., 2006, 2008; Chatburn et al., 2013). Other studies used a slightly different *ad-hoc* division of sigma power into slow (11.5–12.5 Hz) and fast (13.5–14.5 Hz) sigma bands (Bang et al., 2014). Only a handful of studies relied on individually determined spindle frequencies, either by using the IAM method (Bódizs et al., 2005, 2008; Ujma et al., 2014) by computing individual relative sigma power defined as power ± 2 Hz around a single maximal spectral peak relative to the background EEG (Gottselig et al., 2002; Geiger et al., 2011) or by using an adaptive, probabilistic method (Nonclercq et al., 2013).

In sum, our results show that in case of fast spindles, duration and amplitude can be estimated reliably with both fixed and individual frequency methods. Much less consistency can be reached in case of slow spindles, and fixed cutoff frequencies may also lead to a poor separation of slow and fast spindles. Our results suggest that the cutoff frequencies and bandwidths for slow and fast spindles must be selected carefully and individually determined frequency bands should be considered.

It is notable that the concordance between the two methods is generally highest on typical spindle locations (frontal electrodes for slow spindles and centro-parietal electrodes for fast spindles). Concordance is usually lowest on temporal leads, but remains relatively high in occipital leads, in line with the relatively high spindle amplitude on these electrodes reported in the same dataset (Ujma et al., 2014). Lead-specific findings suggest that the lack of concordance between different spindle detection algorithms is especially problematic when non-prominent (e.g., temporal) leads are investigated.

**Figure 3:**
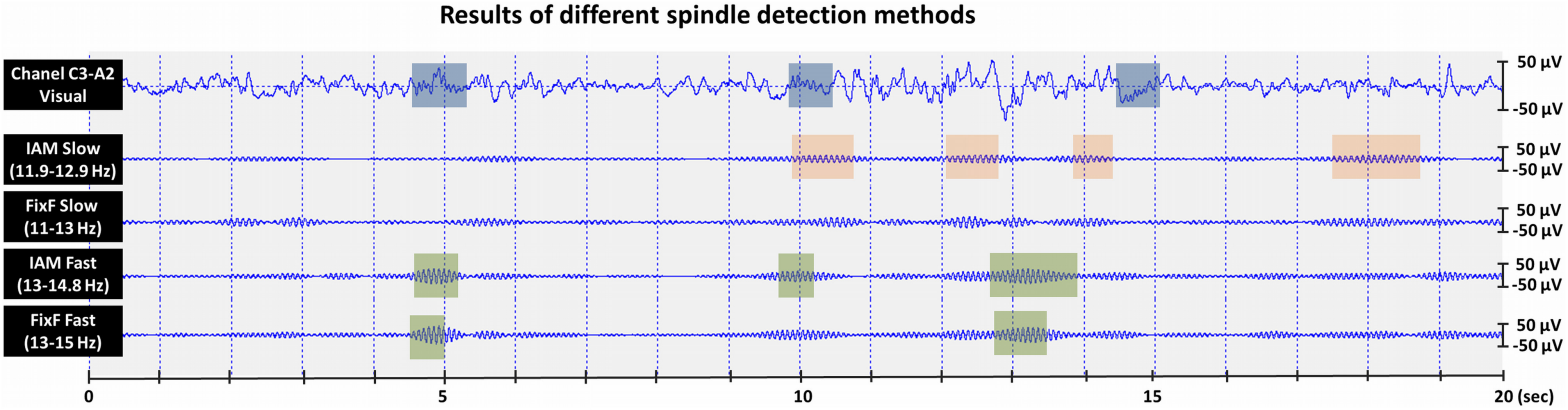
**Visual and automatic sleep spindle detections in a 20 s N2 sleep segment recorded from a healthy adult male**. Unfiltered EEG with the visual detections of an expert rater are shown in the top data line. Appropriately filtered EEG with automatic (IAM and FixF) slow/fast spindle detections are shown in the bottom 4 data lines.

There are limitations of our study that must be mentioned. First, the technical standards of the American Academy of Sleep Medicine (2007) are not met in several subsamples of our study. That is, the analog-to-digital conversion rate is low (8 bits) in the largest subsample (*N* = 114), while the sampling rates are close to the minimally required values (249 and 250 Hz) in two subsamples. Second, while the study compared methodically well-established methods with previous practical applications in science, it must be acknowledged that IAM and the FixF algorithm operate with different philosophical underpinnings, they are designed to detect different features: the FixF method considers the background-relative amplitude of the filtered signal as the key feature of a spindle event, while IAM looks for an amplitude threshold based on the inflection points of the individual EEG spectrum (IAM and FixF detections, together with visual detections are illustrated on Figure 3). Therefore, a perfect agreement between their results cannot be expected, and in the absence of a “gold standard” the inherent superiority of any method cannot be ascertained.

### Conflict of interest statement

The authors declare that the research was conducted in the absence of any commercial or financial relationships that could be construed as a potential conflict of interest.
