# Corrigendum: A comparison of two sleep spindle detection methods based on all night averages: individually adjusted vs. fixed frequencies

**DOI:** 10.3389/fnhum.2015.00415

**Published:** 2015-07-21

**Authors:** Péter P. Ujma, Ferenc Gombos, Lisa Genzel, Boris N. Konrad, Péter Simor, Axel Steiger, Martin Dresler, Róbert Bódizs

**Affiliations:** ^1^Institute of Behavioral Science, Semmelweis UniversityBudapest, Hungary; ^2^Department of General Psychology, Pázmány Péter Catholic UniversityBudapest, Hungary; ^3^Centre for Cognitive and Neural Systems, University of EdinburghEdinburgh, UK; ^4^Max Planck Institute of PsychiatryMunich, Germany; ^5^Department of Cognitive Sciences, Budapest University of Technology and EconomicsBudapest, Hungary; ^6^Nyírõ Gyula Hospital, National Institute of Psychiatry and AddictionsBudapest, Hungary; ^7^Donders Institute, Radboud University Medical CentreNijmegen, Netherlands

**Keywords:** sleep spindles, EEG, individual adjustment method, IAM, algorithm

The description of the Individual Adjustment Method (IAM) algorithm for sleep spindle analyses (Ujma et al., 2015) contained an error, which we hereby rectify. On page 5, line 7–8, instead of f(x) = e^∧^(−(x−xm)/(w/2)), the equation should read as follows:

f(x) = e^∧^−(((x−xm)/(w/2))^∧^2)

## Conflict of interest statement

The authors declare that the research was conducted in the absence of any commercial or financial relationships that could be construed as a potential conflict of interest.
